# A CRISPR design for next-generation antimicrobials

**DOI:** 10.1186/s13059-014-0516-x

**Published:** 2014-11-08

**Authors:** Chase L Beisel, Ahmed A Gomaa, Rodolphe Barrangou

**Affiliations:** Department of Chemical and Biomolecular Engineering, North Carolina State University, Raleigh, NC 27695-7905 USA; Chemical Engineering Department, Faculty of Engineering, Cairo University, Giza, 12613 Egypt; Department of Food, Bioprocessing and Nutrition Sciences, North Carolina State University, Raleigh, NC 27695-7624 USA

## Abstract

Two recent publications have demonstrated how delivering CRISPR nucleases provides a promising solution to the growing problem of bacterial antibiotic resistance.

## The problem(s) with antibiotics

Once the beacon of modern medicine, antibiotics now threaten to be its undoing. These miracle molecules were originally heralded for their remarkable ability to cure a myriad of microbial infections. However, their overuse in medicine and abuse in animal agriculture has led to the rise of multidrug-resistant pathogens that are increasingly tolerant to our current antibiotic arsenal. Even worse, these same antibiotics indiscriminately kill beneficial bacteria along with the pathogens. The consortia of indigenous residents occupying our internal and external bodily surfaces - our microbiome - have been widely implicated in human health, and their disruption by antibiotics is thought to have equally devastating effects. Accordingly, there is a need for novel antimicrobials that can bypass common modes of multidrug resistance while being selective for individual strains. Two recent papers in *Nature Biotechnology* by Bikard *et al*. [1] and Citorik *et al*. [2] offer a promising solution to the problem of antibiotic resistance by using CRISPR (‘clustered regularly interspaced short palindromic repeats’)-Cas (‘CRISPR associated’) systems.

**Figure 1:**
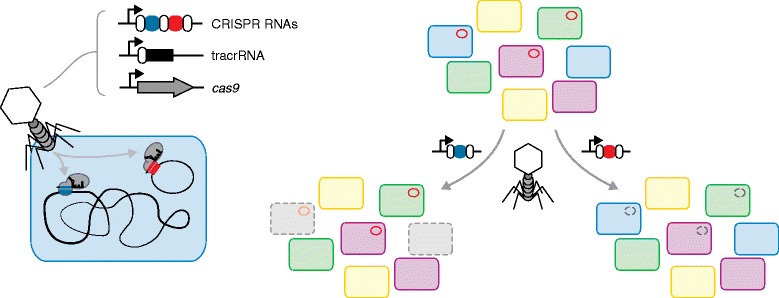
**Delivering CRISPR-Cas9 for targeted killing and plasmid removal.** Left: phages are engineered to encode the Cas9 nuclease, a trans-activating crRNA (tracrRNA) and an array of plasmid-targeting or genome-targeting CRISPR RNAs. The CRISPR RNAs are designed to target unique sequences in the bacterial chromosome or in harbored plasmids. Right: injection of the phage DNA into a mixed population of bacteria leads to removal (here depicted with broken lines) of targeted strains or plasmids without impacting the rest of the population. With further development, this strategy has the potential to treat multidrug-resistant infections without impacting beneficial microbes, to remove contaminating microbes from industrial fermentations and to provide further insights into microbial communities.

CRISPR-Cas systems are adaptive immune systems native to bacteria and archaea that employ CRISPR RNAs to recognize and destroy complementary nucleic acids (Figure 1) [3]. The discovery of one type of CRISPR-Cas system that requires only a single protein for CRISPR-RNA-directed DNA binding and cleavage (Cas9) quickly led to numerous applications, the most popular of which has been genome editing [4]. However, less explored is the potential of these systems to serve as sequence-specific antimicrobials. Early work demonstrated that CRISPR-Cas systems are cytotoxic following incidental self-targeting of the bacterial genome and that they can be used to immunize cells against the spread of multidrug-resistant plasmids [5-7]. Original work from the Marraffini group even suggested that CRISPR-Cas systems could be used for the sequence-specific killing of bacteria [8]. Subsequently, we recently reported the concept of CRISPR-Cas systems as programmable antimicrobials [9], demonstrating that both heterologous and endogenous systems could selectively kill bacterial species and strains. Intriguingly, every sequence in the genome that was targeted led to killing, suggesting that virtually any genomic location could be a distinct target for CRISPR-based antimicrobials [9]. However, an appropriate delivery vehicle was lacking. Now, Bikard *et al*. [1] and Citorik *et al*. [2] tackle this challenge as the next major step towards deploying CRISPR-Cas systems as antimicrobial agents.

## CRISPR-Cas9 to go

For delivery, both studies employed phagemids - plasmids with phage packaging signals - equipped with sequences encoding the *Streptococcus pyogenes* Cas9 nuclease, a designed CRISPR RNA and a trans-activating crRNA (tracrRNA) for CRISPR RNA processing [10]. The beauty of this approach is that phages have already evolved to inject their genetic material into the host bacterium.

The difference between the studies was that Bikard and colleagues [1] used *Staphylococcus aureus* and its temperate phage ϕNM1, whereas Citorik and colleagues [2] used *Escherichia coli* with its filamentous phage M13. Both species are clinically relevant because of their documented antibiotic resistance - particularly multidrug-resistant *S. aureus* (MRSA). The attraction of the phagemid approach rather than use of the phage itself was that new CRISPR RNA sequences could be readily cloned into the phagemid backbone. The packaged phagemids were then employed to target the genome, which led to extensive and rapid killing upon application of increasing amounts of the packaged phagemid. The phagemids were also employed to target harbored antibiotic-resistance plasmids, which led to efficient plasmid removal. Surprisingly, in the study by Citorik *et al*. [2], plasmid removal induced killing. This was traced to the addiction systems of the plasmid that kill the host cell in the absence of plasmid, offering an indirect benefit of targeting some mobile elements encoding drug resistance. Conjugation was also investigated as a means of delivery [2], although the transfer efficiency was too low to substantially reduce cell counts.

With any antimicrobial, the immediate question is how microbes evolve resistance. Remarkably, the survivors did not circumvent targeting - instead they either did not receive the CRISPR-Cas system, or they received a defective system, which is in line with previous findings [9]. The consistency of these findings would argue against the emergence of resistance to CRISPR-Cas-mediated targeting. Instead, other bottlenecks are likely to thwart effective targeting, as will be described later in this article.

Another powerful demonstration of the potential of this technology utilized mixed bacterial communities. The authors relied on two-member or three-member communities of genetic variants of the same strain - a step towards natural communities. In both cases, the authors could specifically eliminate individual target strains while sparing non-target strains. Citorik and colleagues were able to distinguish a single base-pair change between two of the strains, underscoring the specificity of targeting. By exploiting the multiplexable nature of CRISPR, the authors also demonstrated that the CRISPR RNAs also could be readily arrayed to concurrently target more than one strain or plasmid at a time.

To further extend their results, both studies conducted *in vivo* experiments. Bikard *et al*. [1] employed a skin infection model in mice with a co-culture of one targeted fluorescent strain and one non-targeted non-fluorescent strain of *S. aureus*. Citorik *et al*. [2] employed an infection model in which larvae of the honeycomb moth *Galleria mellonella* were fed enterohemorrhagic *E. coli* (EHEC) O157:H7. In both cases, application of the phagemids had a modest but statistically significant effect on the target strain - either by reducing the fraction of fluorescent *S. aureus* strains occupying the skin of the mouse or by improving the survival of the flat worms. While there is room for improvement, these findings offer the first step towards the *in vivo* delivery of CRISPR-Cas systems in clinical and environmental settings.

## The path forward

These initial demonstrations open a wide range of applications for the delivery of CRISPR-based antimicrobials that are otherwise poorly addressed by traditional antibiotics. The primary focus of these studies was treating multidrug-resistant infections without compromising the normal flora, either by killing the pathogen or by restoring its susceptibility to antibiotics. However, many more opportunities exist. For instance, these technologies might be used to study natural and synthetic microbial communities, ranging from those populating our digestive tracts to those in the soil. Engineered phages could partially or completely remove individual members in order to study how the whole community responds over time. Separately, engineered phages could clear heavily guarded niches. By opening these niches, beneficial or diagnostic strains could be administered to take hold of the niche and establish long-term residency in the community. A third opportunity is using these phages to prevent the spread of multidrug-resistance markers in natural environments, thereby stymying the further dissemination of resistance. Finally, eliminating contamination of batch fermentations without compromising the production host could combat a common and economically costly industrial problem. New ways of addressing this issue without discarding the batch could be a major financial boon across the food, beverage, biotechnology and therapeutic industries.

With these applications in mind, a major question is whether use of lytic phages themselves would be sufficient for the same end. Lytic phages are normally strain-specific, replicate as part of the killing process, can be readily isolated from the environment and do not necessarily require any genetic modification. Indeed, lytic bacteriophages are being actively explored as a means of combating multidrug-resistant infections and food contamination. One unique opportunity is incorporating CRISPR-Cas9 into lysogenic bacteriophages, which would greatly expand the set of phages that can be employed as antimicrobials. Another opportunity is using CRISPR-Cas9 to target features that distinguish otherwise-identical strains, such as a recently acquired antibiotic-resistance gene. Finally, CRISPR-Cas9 can be readily programmed to target different species, whereas a new lytic phage would need to be isolated and characterized.

## Hurdles ahead

To truly exploit the capabilities of CRISPR-Cas9, delivery vehicles are needed that can inject their cargo into diverse strains. Broad-host-range phages are extremely rare, and those that are known, at best, infect species within a single genus. Despite phages serving as the first model system in molecular biology, little is known about how to alter or expand their host range. We see this as an excellent opportunity to interrogate poorly understood elements of phage biology while generating phages that can infect virtually any host microbe. Alternatively, nanoparticles or outer-membrane vesicles offer additional promising, yet poorly explored, delivery options.

Using such broad-spectrum delivery vehicles, or any delivery vehicle for that matter, poses a number of challenges that will impact the efficacy of the approach. As evident in these two papers, efficacy dropped substantially in the relatively simple *in vivo* experiments. The first challenge is that the vehicle needs to reach the site of infection in sufficient numbers to deliver the cargo into all possible strains. In natural communities such as the gut microbiota, this would require the particles to survive ingestion and reach the approximately 100 trillion cells of the digestive tract in locations of varying accessibility, which is a formidable challenge. A second challenge is that appropriate surface receptors would need to be expressed on the cells for phage infection - expression levels of these receptors can vary across the population, depending on the environmental conditions. Third, once injected into the cell, the DNA must bypass the defense systems of the host (for example, restriction-modification systems, native CRISPR-Cas systems) and lead to sufficient expression of CRISPR-Cas9. Finally, the targeting sequence must be carefully selected to avoid incidental killing of other strains, although advances in next-generation sequencing are providing a wealth of data for identifying appropriate sequences. Going forward, further efforts will need to tackle each of these barriers. However, each challenge should be surmountable, potentially yielding versatile tools to study and remodel microbial communities as well as providing tailored antimicrobials for the treatment of multidrug-resistant infections.

## Abbreviations

Cas: CRISPR associated
CRISPR: clustered regularly interspaced short palindromic repeats
EHEC: enterohemorrhagic *E. coli*
MRSA: multidrug-resistant *S. aureus*
tracrRNA: trans-activating crRNA
